# Why work-related causes and causal attributions should be assessed separately from depressive symptoms: Response to Bianchi and Schonfeld

**DOI:** 10.1177/10519815251344887

**Published:** 2025-05-25

**Authors:** Maie Stein, Hannes Zacher

**Affiliations:** 1 Wilhelm Wundt Institute of Psychology, Leipzig University, Leipzig, Germany

**Keywords:** depression, measurement, occupation, strain, work stress, causal attribution

## Abstract

In this response to Bianchi and Schonfeld's rebuttal to our commentary on the Occupational Depression Inventory (ODI), we further elaborate on our conceptual and methodological arguments for why the conflation of depressive symptoms with their attributed work-related causes in the ODI is problematic for its construct and criterion-related validity. Although we acknowledge the relevance of considering people's beliefs about the work-related causes of depression, we emphasize the importance of clearly distinguishing between depressive symptoms, their potential causes, and causal attributions in research that aims to examine the antecedents and consequences of depression in the work context. Based on a causal attribution model of depression in the work context, we illustrate how the conflation of depressive symptoms with their attributed work-related causes in the ODI introduces interpretive challenges in both cross-sectional and longitudinal research. We recommend that future research employs separate measures to assess depressive symptoms, their potential work-related causes, and causal attributions to refine our understanding of the complex relations among these constructs and to provide insights into their distinct antecedents and consequences.

We have recently pointed out that, like many other measures of job strain,^1–3^ the Occupational Depression Inventory (ODI)^4,5^ is a confounded measure, as it conflates depressive symptoms with their attributed work-related causes. In our commentary, we presented several reasons why this confounding undermines the construct and criterion-related validity of the ODI.^6^ In their rebuttal, Bianchi and Schonfeld have largely dismissed these concerns and suggested that we “mischaracterize” their measure.^7^ In this response, we further clarify, refine, and extend our conceptual and methodological arguments.

We agree that “the issue of confounding […] is nothing new in the context of measures involving causal attributions”,^7^ and we acknowledge that including work-related causal attributions was an intentional design feature of the ODI.^4,5^ We also recognize the need for empirical insights into how respondents interpret the ODI item on suicidal ideation (“I thought that I’d rather be dead than continue in this job”), and whether this item conflates metaphorical expressions of work-related frustration with literal suicidal intent. Addressing this issue requires carefully designed qualitative studies on interpretive processes (e.g., cognitive interviewing), as well as epidemiological studies comparing general vs. work-related suicidal ideation. Nonetheless, we maintain that this item is subject to the same conceptual and methodological issues of confounding as the rest of the ODI, as it conflates a depressive symptom with the attribution of work as its cause.

We agree with the ODI authors that considering people's beliefs about the work-related causes of depressive symptoms is important. Indeed, causal attributions can shape cognitions, affect, and behavior beyond the impact of the experience itself.^8^ For example, the attributional model of work exhaustion consequences^9^ posits that workers’ beliefs about the causes of their exhaustion can independently influence important work outcomes, such as job satisfaction, commitment, and turnover.

Consistent with this model, we argue that research on the antecedents and consequences of depression in the work context should conceptually distinguish depressive symptoms, their potential work-related causes, and individuals’ attributions regarding these causes. Methodologically, these constructs should be assessed separately to capture their complex relations and distinct – both additive and interactive – effects on work-related cognitive-affective reactions and behaviors. To illustrate this point and highlight the conceptual and methodological issues of incorporating work-related causal attributions directly into depression measures, we adapt the attributional model of work exhaustion consequences^9^ to the domain of depression in the work context (see Figure 1).

**Figure 1.**
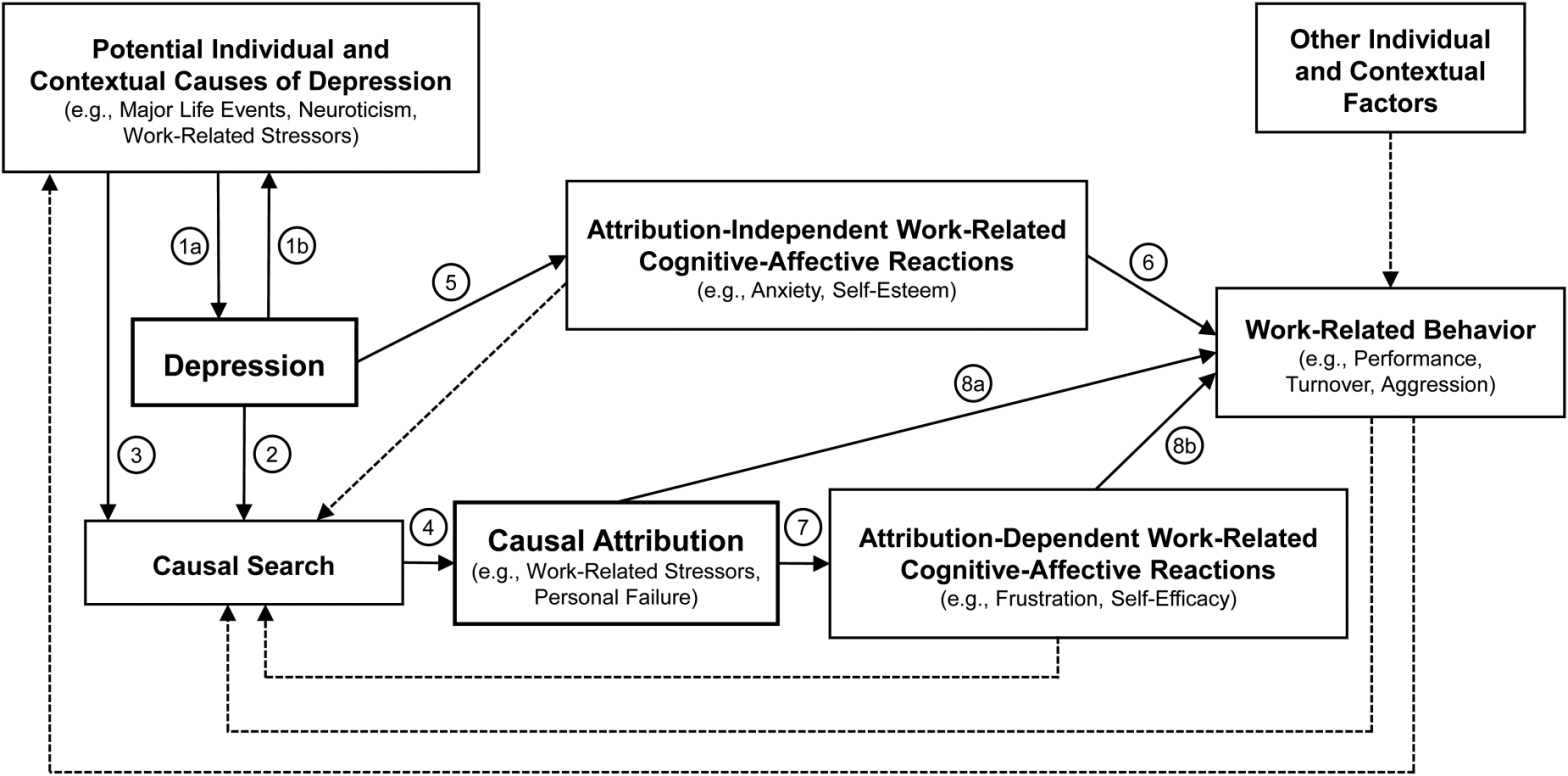
Causal attribution model of depression in the work context.

*Note*. This model was adapted from Moore.^9^ The dashed lines represent paths that are not the main focus of our model.

Based on research on the etiology of depression,^10,11^ our adapted model posits that depression can be caused by various individual and contextual risk factors (as well as their interactions), including work-related stressors^12^ (Path 1a in Figure 1). In response to depressive symptoms, individuals may attempt to make sense of their experience by trying to identify its causes^13^ (Path 2). This causal search is shaped by the perceived presence and salience of potential causes (Path 3) and may lead to causal attributions (Path 4). In this process, individuals may – or may not – attribute their symptoms to work-related factors.^14^

The model further posits that depressive symptoms and causal attributions influence work-related cognitive-affective reactions and behaviors through distinct pathways. On the one hand, depressive symptoms may elicit attribution-independent work-related cognitive-affective reactions (Path 5), such as increased anxiety and reduced self-esteem,^15^ which, in turn, may decrease favorable behaviors, such as work performance, and increase unfavorable behaviors, such as turnover (Path 6).^16,17^ On the other hand, attributing symptoms to work-related causes may elicit attribution-dependent work-related cognitive-affective reactions (Path 7), such as increased frustration, which, in turn, may lead to unfavorable behaviors, such as aggression^18^ (Paths 8a and 8b).

This model illustrates that conflating depressive symptoms and their attributed work-related causes within measures of depression in the work context introduces ambiguity. It becomes unclear whether the measure captures depressive symptoms, work-related stressors, or beliefs about work as the cause of depression (The ODI does not clearly distinguish between work-related stressors and work-related strain and also refers to work stress more broadly, which limits insights into the distinct roles of (perceived) external conditions (i.e., work-related stressors) and internal experiences (i.e., work-related strain), as well as the overall process of work stress (i.e., stressors predicting strain), in shaping workers' beliefs about the causes of their symptoms). This ambiguity is compounded by evidence suggesting that work-related stressors (e.g., illegitimate tasks, organizational injustice) and strain (e.g., depression) are reciprocally related over time^19–21^ (Path 1b). As such, individuals may attribute depressive symptoms to work because work has actually caused them, but depressive symptoms may also influence how individuals perceive their work environment. Supporting this latter possibility, research has shown that workers with higher levels of depression are more likely to interpret ambiguous job-related information negatively.^22^

Importantly, we did not aim to “mischaracterize” the ODI, nor do we recommend that it “should be discarded”.^7^ The appropriateness of any measure depends on its intended use. For example, the ODI may be appropriate for estimating the prevalence of “occupational depression,” provided that there is a clear understanding that this construct inherently involves causal attributions to work-related factors, and that it is recognized that the resulting data does not reflect causal relations between work-related stressors and depressive symptoms. We also agree that the measure may yield practically relevant insights into individuals’ beliefs about the causes of their symptoms, provided that it is acknowledged that the search for such causes is a cognitively challenging process, especially for individuals with depression, and that people do not always accurately identify the causes of their symptoms.

However, we maintain that using the ODI to examine the antecedents and consequences of depression in the work context is problematic, as the conflation of symptoms with attributed causes undermines the interpretability of any associations found. The ODI authors suggest that this issue is primarily problematic in cross-sectional research.^7^ However, the confounding also limits the interpretability of findings from longitudinal studies. When symptoms and attributions are combined into a single measure, changes over time are difficult to interpret: Do they reflect changes in symptom severity, in work stressors, in causal attributions, or some combination? Additionally, since the ODI captures perceptions of work stress, it conceptually – and likely empirically – overlaps with measures of work-related stressors. In longitudinal studies, this overlap may obscure relations between work-related stressors and depressive symptoms. Scholars may erroneously conclude that specific work-related stressors do not predict depression, when in fact, shared variance between stressor measures and the ODI masks these relations.

In conclusion, we recognize the strong psychometric properties of the ODI and its potential value in certain practical applications. However, we uphold our concern that conflating depressive symptoms with their attributed work-related causes can lead to erroneous conclusions about the antecedents and consequences of depression in the work context, both in cross-sectional and longitudinal studies. To advance the understanding of the complex relations among depression, work-related stressors as potential causes of depression, causal attributions, and work-related cognitive-affective reactions and behavior, we encourage future research to refine and test our proposed conceptual model (Figure 1) using separate measures of these constructs.
